# Body and Boat: Significance of Morphology on Elite Rowing Performance

**DOI:** 10.3389/fspor.2020.597676

**Published:** 2020-12-02

**Authors:** Quentin De Larochelambert, Scott Del Vecchio, Arthur Leroy, Stephanie Duncombe, Jean-Francois Toussaint, Adrien Sedeaud

**Affiliations:** ^1^EA7329, Institute for Research in BioMedicine and Epidemiology of Sport (IRMES), Paris, France; ^2^MAP5 — Université de Paris, Paris, France; ^3^CIMS, Hôtel-Dieu, Assistance Publique—Hôpitaux de Paris, Paris, France

**Keywords:** rowing, pacing, morphology, performance, height, anthropometry

## Abstract

**Objectives:** The purpose of this study was to determine and weigh the anthropometric indicators that were associated with pacing performances for each Olympic rowing category.

**Methods:** Between 2010 and 2015, 1,148 rowers (650 men and 498 women) participated in the finals of World Championships in each heavyweight Olympic event. They were categorized into four morphological clusters according to their height and body mass index (BMI): tall and thin (TT), tall and robust (TR), small and thin (ST), and small and robust (SR). Time and speed, were collected every 50 m for all boats in each competition. Non-parametric inferential methods were used to understand the differences in performance between morphological clusters over the entire race. After, we calculated a new indicator to determine the differences between these morphotypes within the race.

**Results:** In this article, we determined which morphologies had a significant effect on speed for both men and women. For example, the biggest rowers were the fastest in skiff. Analysis of each 50 m demonstrated that between the four morphological categories that the TR male athletes were significantly faster than their ST counterparts between the 800 and 2,000 m of the race by 1.76% of mean speed. Furthermore, the SR were the fastest in female coxless pairs over the majority of the race. These differences in speed by morphological cluster are summarized, by race segment, for all categories and sex.

**Conclusion:** Anthropometric factors impact pacing among rowers' categories. Coupling anthropometry and race pacing is not only helpful to understand which factors work where, but is also helpful in improving training and performance. This can help both in the recruiting of rowers for specific boats and adapting the race strategy. In future, the method used can be adapted for factors other than anthropometry. It can also be individualized to enable athletes to prepare for their race according to future competitors.

## Introduction

Besides physiological, biomechanical, and psychological properties, an athlete's profile is also based on an anthropometric foundation for human, and specifically rowing, performance (Bourgois et al., 2000, 2001). Morphology in elite sport whether for identification or training can be useful, although potentially deceitful. Using all available performance parameters can help narrow down which ones are useful for determining what type of athlete is optimal for elite rowing teams and championship crews. There are many simple biometric factors that provide a great deal of information about the athlete (Sedeaud et al., 2012, 2014b). In athletics, height and mass are linked to speed: for sprinters “heavier and taller is better” and “lighter and smaller” runners are better in endurance events (Sedeaud et al., 2014a). Some rowing studies have investigated morphological traits, especially the factors that impact performance the most, including standing height, mass, lean body mass, and leg length (Shephard, 1998). Increased body mass (Secher and Vaage, 1983; Shephard, 1998) and body size (Hebbelinck et al., 1980; deRose et al., 1989) have shown to be positive for rowing performance. Unfortunately, the relationship between speed pacing during races and morphological traits is still vague.

Moreover, there is little research on the correlation between morphological traits and race pacing. Race pace studies are useful for observing the speed of the crews during races, but currently only use intervals of 500 m. A parabolic-shaped velocity curve during racing is observed (Secher, 1983). With this foundation, more recent studies have shown that there are no significant differences in race pace distributions between winners and losers as well as men and women (Garland, 2005; Muehlbauer and Melges, 2011). Those studies were limited by distance measurements that were only available per 500 m. Based on these relationships, rowing falls between two types of racing: sprinting and long-distance. Sprinting in cycling and track starts at a high pace and then remains even, while long-distance events follow a negative split pattern (de Koning et al., 1999). Because of the difference in the energy system used, rowing does not follow any of those patterns. According to Muehlbauer and Melges, single boats are better described by a linear trend line with a positive slope, whereas a linear trend line and a quadratic trend line better described all other multi-person boats (Muehlbauer and Melges, 2011). That same study also showed a difference of in-race variances between the heats and the final. Heats follow a linear trend line while finals followed a quadratic trend.

In addition, only a few of the current studies include elite athletes. A protocol on Olympic rowers highlighted the anthropometric and physiological profiles of Croatian rowers. It showed that the best athletes had higher values of segmental lengths, circumferences, and muscle widths (Mikulić et al., 2007). Furthermore, their oxygen consumption and power output at anaerobic threshold were higher (Mikulić et al., 2007). In addition, one study was interested in the influence of kinetic and dynamic variables measurable in real conditions on the speed of the boat (Perić et al., 2019) but only in few athletes (*n* = 12). It highlighted that the speed of the boat is mostly correlated with rowing power, finishing angle and average force. It also showed that elite rowers have higher mean values for two additional dynamic variables (work per shot and maximum strength) and two anthropometric variables (body weight and body size) in comparison to sub-elite rowers. These anthropometric indicators, along with Body Mass Index (BMI), have also been studied to assess their influence on career level (Winkert et al., 2019). This study of 910 former rowers of the German National Junior Team showed that height and body mass affect the level of career in men. However, this study evaluated the influence of anthropometric traits only on the long-term level.

Several studies have attempted to analyze performance longitudinally using different statistical models. One study (Thibault et al., 2010) created an algorithm allowing the comparison of several performance trends (world record) of various Olympic disciplines (Track and Field, Swimming, Cycling, Speed, Skating, and Weightlifting) for more than 50 years. They also use an inferential method (Wilcoxon test) to determine the significance of the difference. Other studies (Moore, 1975; Berthelot et al., 2012, 2019) use two nonlinear regressions to theorize the relationship between age and performance in many sports disciplines.

To the best of our knowledge, there is no study that contains all three areas of research: anthropometric traits, pacing, and elite athletes. The method of combining human indicators such as height, mass, and BMI with race pace factors can allow for a better understanding of the best performing rowers and crews over the past World Championships. The purpose of this study is to determine and weigh the anthropometric indicators that are related to pacing performance for each Olympic rowing category and sex. The main hypothesis is that body type has a significant impact on rowing performance but is dependent on the race category and sex of the athletes.

## Methods

### Sample

#### Inclusion Criteria

An athlete that has competed in either an A or B final at the World Championship between 2010 and 2015 in a heavyweight category (M1x, W1x, M2−, W2−, M2x, W2x, M4−, M4x, W4x, M8+, and W8+).

#### Exclusion Criteria

An athlete on a boat with inaccessible speed data or an athlete with unavailable anthropometric data.

### Data Collection

There were 1,148 rowers eligible for inclusion (650 men and 498 women), which totaled 2,120 performances. Variables collected included their standing height (cm), body weight (kg), which final they competed in, their rank in the final, as well as their country and category. Time and speed were collected per 50 m for all boats. All anthropometric and performance data were collected on the site http://www.worldrowing.com/. Speed and time data per 50 m were obtained using GPS tracker. Anthropometric data were self-reported by athletes during their competitions.

### Study Design

This is an open cohort study involving the top 12 crews in each Olympic category. This dynamic population of elite rowers constantly changes from year to year, but mainly includes the same top rowers throughout an Olympic cycle. Those rowers occasionally change events or can continuously go in or out of our cutoff of 12th place.

### Data Analysis

#### Classification of Morphotypes

With all the collected data, we calculated BMI for each athlete and an average for their respective crew.

Using all this information, we separated each rower or crew into four groups of equal sizes, based on anthropometry, as follows:

- Tall and Robust (TR): top 50% Height / top 50% BMI
- Tall and Thin (TT): top 50% Height / bottom 50% BMI
- Small and Robust (SR): bottom 50% Height / top 50% BMI
- Small and Thin (ST): bottom 50% Height / bottom 50% BMI.

These groups were then used to stratify boats and assess race pace each 50 m by group. To study pacing patterns, we introduced two indicators to exhibit differences between the four morphological clusters.

#### Influence of the Morphotype on Performance

For each category, the Kruskal Wallis non-parametric test was carried out on the speed of races between the four morphological clusters, to determine if the morphology had an impact on the average speed. If the previous test was significant, a post hoc Wilcoxon test with Bonferroni adjustment was performed to compare each of the morphological clusters. If the post hoc test revealed a significant difference between two categories, two indicators were calculated.

- The first indicator was the percentage of speed gain from a morphological cluster over another one.
- The second indicator was the maximal interval of race meters on which one group lead another, this last piece of information shows the main part of the race where there is domination from a morphological cluster, in the ambiguous case where the two curves are mixed up.

The theoretical details allowing us to define these indicators follow below. The number of traveled race meters as *x*∈[0, 2000]. For each value of x, we associate the function *f*_*Ci*_(*x*), which is the instant speed when rowers have traveled x meters of the race, with *Ci, i*∈{TR, TL, SR, SL} corresponding to the four morphological clusters.

**Part 1:**

To compare the two functions *f*_*Ci*_*and f*_*Cj*_, we compute the area *A*(*Ci, Cj*) between the two corresponding curves:

$$\begin{array}{l} A\left(Ci,Cj\right)=\underset{0}{\overset{2000}{\int }}f_{Ci}\left(x\right)-f_{Cj}\left(x\right)dx \end{array}$$

It follows with the mean speed difference that is given by

Considering that our data are records of speed per 50 m, we discretized the interval as 40 regular parts of 50 m and computed corresponding sums to get for each *i, j*∈{TR, TL, SR, SL}.

In order to interpret in an easier way this difference, we will express this quantity as the percentage of speed difference Δ*per*(*Ci, Cj*) between *CiandCj*. Therefore, we used the appropriate standardization:

$$\begin{array}{l} \Delta per\left(C_{i},C_{j}\right)=\frac{\Delta \left(C_{i},C_{j}\right)}{\underset{0}{\overset{2000}{\int }}f_{Cj}\left(x\right)dx}.100 \end{array}$$

**Part 2:**

We then defined the maximal interval in which *f*_*Ci*_ > *f*_*Cj*_, *Imax*(*Ci, Cj*).

$$\begin{array}{l} Imax\left(Ci,Cj\right)=\;\underset{\left[a,b\right]}{\max}\left(\forall x\in \left[a,b\right],\;f_{Ci}\left(x\right)>\;f_{Cj}\left(x\right)\right) \end{array}$$

## Results

### Overall Results

**Figure 1:**
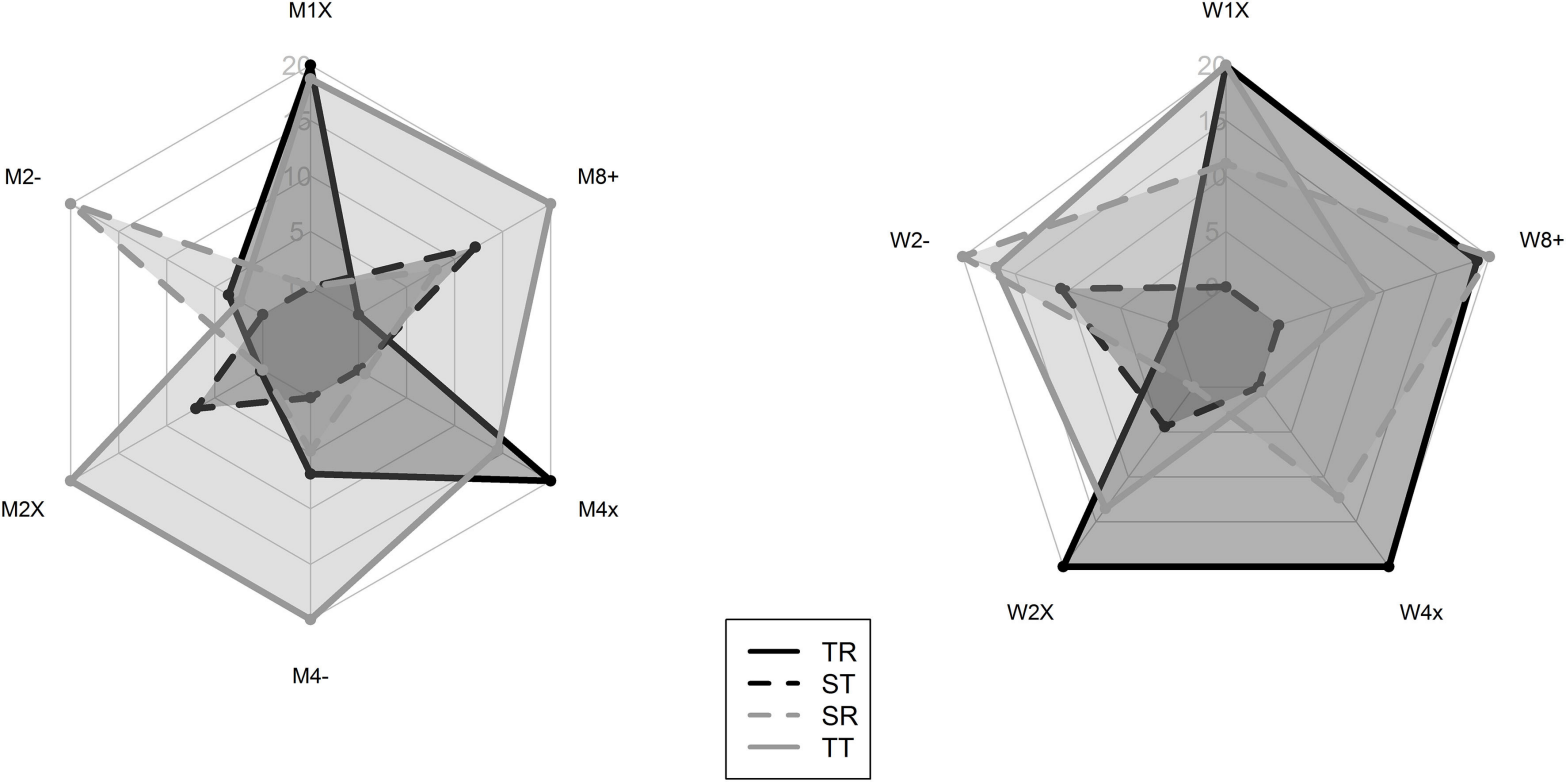
Performance comparison of morphological clusters for each crew in male **(left)** and female **(right)** rowers.

**Figure 2:**
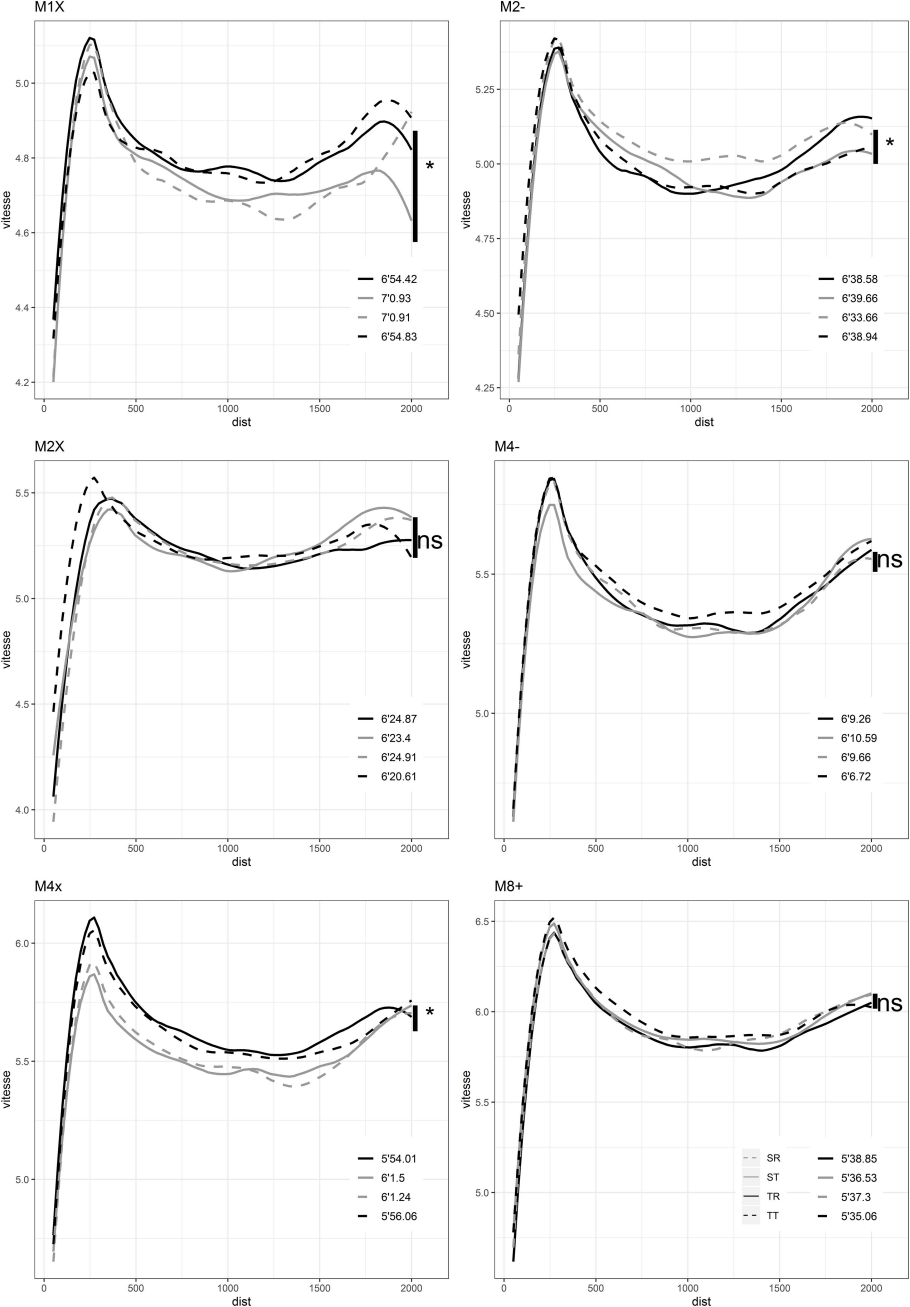
Race pacing according to morphological categories for men.

**Figure 3:**
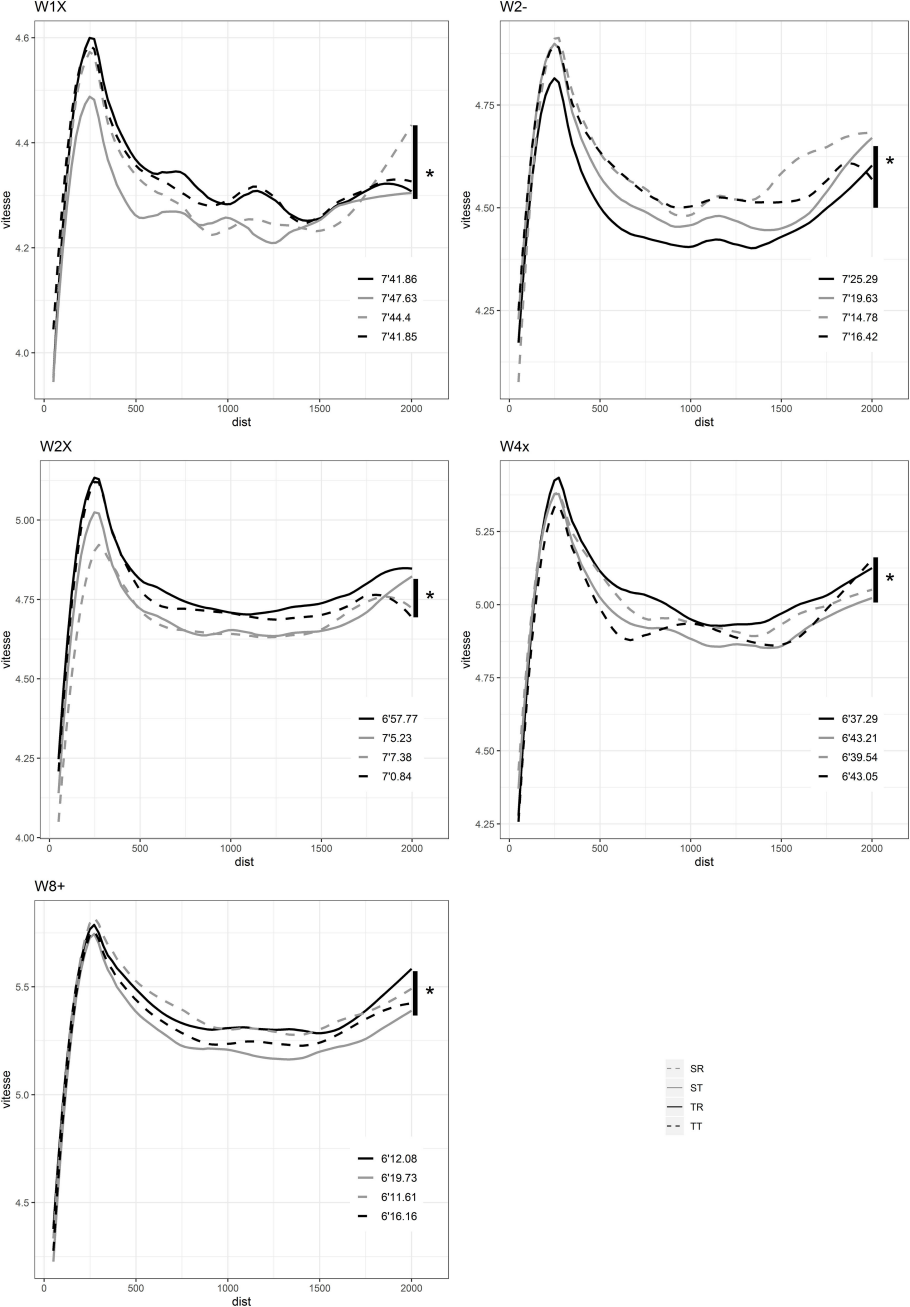
Race pacing according to morphological categories for women.

The total area in Figure 1 represents the overall success of each morphotype in rowingfor men and women. For example, there is an overall dominance by TT in men's races and TR in women's races which have a larger area than the other morphotypes. ST are slower overall for both male and female rowers (Figure 1). For men, SR are poor overall except in the coxless pair. Performance comparison by morphology for each category are shown in Figures 2, 3 for males and females respectively. The different morphotypes significantly influenced the overall race outcomes for the categories M1x, M2-, M4x, W1x, W2-, W2x, W4x, and W8+ (*p* < 0.05) (Figures 2, 3). Pacing differences existed throughout the race for both sexes in each category depending on the 50 m section analyzed (Tables 1, 2).

**Table 1:** Percentage of speed gain from a morphological cluster over another one and portion of race where a crew was faster in men's rowing.

**Category**	**P1**	**P2**	**P1 > P2**	**% P1 > P2**	**P2 > P1**	**% P2 > P1**
M1X	TR	ST	[800, 2000]	1,76		
M1X	TR	SR	[450, 1950]	1,93	[1950, 2000]	0,48
M1X	TR	TT	[100, 550]	1,45	[1600, 2000]	0,91
M1X	ST	SR	[1150, 1850]	0,72	[300, 450]	1,47
M1X	ST	TT	[200, 350]	0,83	[700, 2000]	1,94
M1X	SR	TT	[200, 450]	1,17	[500, 2000]	2,10
M2-	TR	ST	[1200, 2000]	1,37	[500, 1050]	1,29
M2-	TR	SR	[1850, 2000]	0,65	[100, 1850]	1,45
M2-	TR	TT	[1200, 2000]	1,34	[100, 400]	1,24
M2-	ST	SR			[0, 2000]	1,52
M2-	ST	TT	[800, 1050]	0,82	[100, 450]	1,26
M2-	SR	TT	[300, 2000]	1,73	[100, 200]	1,65
M2X	TR	ST	[150, 800]	1,42	[1150, 2000]	1,66
M2X	TR	SR	[700, 1000]	0,41	[1050, 1450]	0,21
M2X	TR	TT	[700, 1000]	0,49	[1500, 1900]	1,61
M2X	ST	SR	[1200, 1950]	1,03	[250, 800]	0,96
M2X	ST	TT	[1550, 2000]	1,42	[900, 1250]	0,89
M2X	SR	TT	[400, 900]	0,48	[950, 1400]	0,71
M4-	TR	ST	[100, 700]	1,06	[1800, 2000]	1,40
M4-	TR	SR	[1550, 1800]	0,60	[1850, 2000]	0,53
M4-	TR	TT			[200, 2000]	1,04
M4-	ST	SR	[1700, 2000]	0,78	[200, 450]	1,53
M4-	ST	TT			[100, 1850]	1,51
M4-	SR	TT			[1050, 1850]	1,47
M4x	TR	ST	[100, 2000]	2,21		
M4x	TR	SR	[100, 2000]	2,11		
M4x	TR	TT	[1150, 2000]	0,73		
M4x	ST	SR	[1300, 1650]	0,73	[250, 550]	0,77
M4x	ST	TT			[100, 1750]	1,78
M4x	SR	TT			[100, 1800]	1,61
M8+	TR	ST	[750, 800]	0,04	[100, 500]	1,00
M8+	TR	SR	[1050, 1200]	0,63	[1350, 2000]	1,15
M8+	TR	TT	[1950, 2000]	0,06	[100, 1950]	1,15
M8+	ST	SR	[100, 450]	0,97	[1400, 1800]	0,64
M8+	ST	TT	[1850, 2000]	0,37	[300, 1050]	0,77
M8+	SR	TT	[1550, 1700]	0,52	[100, 900]	1,31

**Table 2:** Percentage of speed gain from a morphological cluster over another one and portion of race where a crew was faster in women's rowing.

**Category**	**P1**	**P2**	**P1 > P2**	**% P1 > P2**	**P2 > P1**	**% P2 > P1**
W1X	TR	ST	[150, 1450]	1,76	[1650, 1700]	0,31
W1X	TR	SR	[600, 1400]	1,23	[1800, 2000]	1,10
W1X	TR	TT	[650, 900]	0,79	[100, 200]	0,89
W1X	ST	SR	[1000, 1100]	1,08	[150, 650]	1,78
W1X	ST	TT	[1650, 1700]	0,26	[100, 1450]	1,61
W1X	SR	TT	[1850, 2000]	1,02	[600, 1350]	1,09
W2-	TR	ST			[0, 2000]	1,30
W2-	TR	SR	[100, 150]	0,40	[150, 2000]	2,66
W2-	TR	TT			[100, 2000]	2,17
W2-	ST	SR	[100, 200]	2,12	[350, 2000]	1,56
W2-	ST	TT	[200, 250]	0,18	[400, 1950]	1,03
W2-	SR	TT	[1400, 2000]	1,55	[900, 1150]	0,43
W2X	TR	ST	[0, 2000]	1,82		
W2X	TR	SR	[100, 2000]	2,35		
W2X	TR	TT	[1200, 1750]	0,79		
W2X	ST	SR	[150, 350]	2,99	[1600, 1900]	0,58
W2X	ST	TT	[1900, 2000]	1,98	[100, 1900]	1,26
W2X	SR	TT	[1900, 2000]	0,75	[150, 1550]	1,92
W4x	TR	ST	[150, 2000]	1,63	[100, 150]	0,77
W4x	TR	SR	[1200, 2000]	0,80	[100, 150]	1,96
W4x	TR	TT	[150, 1100]	1,95		
W4x	ST	SR	[250, 300]	0,23	[350, 1350]	1,07
W4x	ST	TT	[550, 900]	0,90	[950, 1250]	1,14
W4x	SR	TT	[100, 1000]	1,50	[1800, 2000]	0,78
W8+	TR	ST	[0, 2000]	2,01		
W8+	TR	SR	[1750, 2000]	0,85	[250, 900]	0,92
W8+	TR	TT	[0, 2000]	1,05		
W8+	ST	SR			[200, 2000]	2,25
W8+	ST	TT			[1000, 2000]	1,20
W8+	SR	TT	[200, 2000]	1,25		

### Morphological Influences on Men Performances

#### M1x

The fastest during the entire race are the Tall and Robust and the Tall and Thin (Figure 2). The specific differences expressed for each 50 m between the four morphological categories are detailed in Table 1. For example, the TR athletes are significantly faster than ST between the 800 and 2,000 m of the race. They are faster by 1.76% of mean speed (*p* < 0.05) and faster than the SR athletes by also 1.93% of speed but between the 450 and 1,950 m (*p* < 0.05). The TT athletes are significantly faster than their ST counterparts by 1.94% of speed during the 700–2,000 part of the race, (*p* < 0.05) and SR by 2.1% of speed, during the 500–2,000 race part, (*p* < 0.05) (Table 1).

#### M2-

The fastest athletes are the small and robust (SR) athletes (Figure 2). The SR athletes are significantly faster than their small and thin (ST) counterparts by 1.52% of mean speed during the entire race, and by 1.73% than their TT counterparts, from 300 m to the end of the race (Table 1). All the other specific differences are detailed in Table 1.

#### M2x

The fastest athletes are the Tall and Thin (TT) athletes (Figure 2). They are faster than their TR counterparts by 1.61% of speed between the 1,500 and 1,900 m (Table 1).

#### M4-

The TT are the fastest in coxless (Figure 2). They are faster than their ST counterparts by 1.51% of mean speed from 100 to 1,850 m (Table 1).

#### M4x

TR are the fastest and ST are the slowest ST (Figure 2). TR are significantly faster than ST by 2.21% of mean speed from the 100 to 2,000 m. TT are significantly faster than SR by 1.61% of speed from the 100 to 1,800 m (Table 1).

#### M8+

Tall and thin (TT) are the fastest in “eight with coxswain” (Figure 2). They are faster than their SR counterparts by 1.31% of speed between the 100 and 900 m (Table 1).

### Morphological Influences on Women's Performances

#### W1x

The TR and TT are the fastest in skiff and the ST are the slowest (Figure 3). TR are significantly faster than ST by 1.76% of mean speed between the 150 m to the 450 m (Table 2). TT are significantly faster than ST by 1.61% of mean speed from 100 to 1,450 m (Table 2).

#### W2-

SR are the fastest in coxless pairs and TR are the slowest (Figure 3). SR are significantly faster than TR by 2.66% of mean speed from 150 to 2,000 and SL by 1.56% of mean speed from 350 to 2,000 (Table 2). SL are significantly faster than TR (1.27%, [0, 2000], *p* < 0.05). TL are significantly faster than TR (1.99%, [50, 2000], *p* < 0.05).

#### W2x

TR are the fastest in coxless pairs and SR are the slowest (Figure 3). TR are significantly faster than ST by 1.82% of mean speed during the entire race and then their SR counterparts by 2.35% from 100 to 2,000 m (Table 2). TT are significantly faster than ST by 1.26%, of mean speed from 100 to 1,900 m (Table 2). TT are significantly faster than SR by 1.92% of mean speed from 150 to 1,550 m (Table 2).

#### W4x

TR are the fastest and ST are the slowest (Figure 3). TR are significantly faster than ST by 1.63% of mean speed from 150 to the end of the race and then TT by 1.95% of mean speed from 150 to 1,100 m (Table 2).

#### W8+

SR and TR are the fastest and ST are the slowest (Figure 3).

SR are significantly faster than ST by 2.25% of mean speed from 200 to the end of the race (Table 2). SR are significantly faster than ST by 2.25% of mean speed from 200 to 2,000 m (Table 2).

## Discussion

This article directly demonstrates the influence of morphology on speed in each rowing category for women's and men's events. This impact may depend on each category in accordance with other studies which demonstrate that bodily dimensions have a significant impact on the speed of the vessel (Bourgois et al., 2000, 2001; Claessens et al., 2005; Mikulić, 2008; Perić et al., 2019).

The fastest in single scull, in men and women, are the TR and the TT. Height seems to be a favorable parameter for performance in this category. We often see a vast set of competitors of single scullers at the World Championships because it is the most attainable event for countries who cannot support many athletes, but by the A and B finals, and especially the podium, the best rowers are the tallest (Secher and Vaage, 1983; Mikulić et al., 2007). It confirms that power and amplitude are preferred in skiff at the expense of velocity and that the strength and power of the rowers are a determining factor in the speed of the boat (Perić et al., 2019). The relationship between speed and morphological group is specific to each category. In elite athletes, bigger morphological traits reveal higher muscle mass, absolute strength and power (Jaric, 2003; Markovic and Jaric, 2004; Sedeaud et al., 2014a). This information when obtained in a boat during competition races is much more useful than on ergometer (Perić et al., 2019). In their study, Perić et al. demonstrated in real conditions that the speed of the boat is correlated with rowing power, work per stroke and maximum force, and two morphological variables. These two anthropometric traits, body mass and height, were significantly higher for the world championship medal winners compared with the sub-elite (Perić et al., 2019). The bigger force of stroke in elite rowers was explain by higher extension in the knee joint (Hase et al., 2004), an ability dependent of height.

In the coxless pair, the SR continually comes out ahead in both women and men. In 2x, the TT athletes are the fastest in men and the TR for women. In men's four and M8+ the TT athletes are the fastest, and the TR row faster for the M4x. For women, the SR rowers are fastest for all boats with four or more athletes involved. Despite boats with multiple rowers were very technical, morphological cluster demonstrate that higher height and mass are useful indicators. Other aspects of performance generate convincing and demonstrated effects such as VO2max, peak power, power resistance, buffering agent, muscle typology, muscle coordination, and biomechanical determinants (Cosgrove et al., 1999; Jürimäe et al., 2000; Baudouin and Hawkins, 2002; Ingham et al., 2002; Bourdin et al., 2004; Janshen et al., 2009; Izquierdo-Gabarren et al., 2010; Forjasz, 2011; Buckeridge et al., 2014), but all these parameters are obviously linked to morphology.

In women, it seems important to have a boat with robust rowers in W8-, in accordance with mass and performance relationships on the ergometer (Yoshiga and Higuchi, 2003). Anthropometric characteristics have been used many times in many different ways throughout studies and have proven to be worthy when helping performance (Sedeaud et al., 2012, 2014a,b) and advising selection processes (Bourgois et al., 2000, 2001). Some authors have concluded that size and mass are among the most important elements of rowing performance: the heaviest and tallest rowers are the fastest (Bourgois et al., 2000; Barrett and Manning, 2004). Moreover, height has changed consequently by 1.6 cm/decade for heavyweight rowers as well as for mass with 1.4 kg/decade increments (Norton and Olds, 2001). Other aspect of results are important, the slowest morphological athletes for M1x, M2^−^, M4^−^, M4x, W1x, W4x, and W8+ are the small and thin one. These data confirm, and are in accordance with, the fact that above-average height and mass should be given advantage for elite rowers and early selection (Perić et al., 2019). These results underline the inherent need of power and cardiopulmonary expressed indirectly by a bigger morphology.

The impact of the morphology on the overall race has already been studied in all race categories (Bourgois et al., 2000, 2001; Claessens et al., 2005; Mikulić, 2008; Perić et al., 2019). The methods in this article have made it possible to differentiate the morphological impacts in particular parts of races. It is well-known that the taller rowers in men's races dominate the skiff. However, the methods developed here have made it possible to highlight that the TR are faster than the TT at the beginning of the race and slower at the end of the race. This allows for the adaption of racing strategies according to anthropometry, which are not visible when the entire race is analyzed together. We also see that in the M8+, where there is a non-significant difference over the entire race between the SR and the ST, that the ST perform better at the start of the race and worse at the end of the race compared to the SR.

These results and the method developed here have large potential for opponent and strategic analysis. The classification method was chosen to be capable of generating the most explicit results and therefore, enabling it to be tangible and understandable for coaches and rowers. Applying these methods, which are able to differentiate the part of the race where there is domination, could be pertinent for opponent analysis. Other interesting uses could be to clarify which areas of the race should be worked on during training, when and where athletes may provide optimal attacks, and to determine what speeds and cadences should be targeted for success, while acknowledging the environmental conditions.

## Limitations

The developed method uses running speed to study differences in performance according to body type. We know that rowing speed depends on many parameters in addition to anthropometry. Therefore, moving forward it would be useful to have additional data to minimize the effect of confounding variables influencing speed. For example, environmental conditions also play a major role in the race. In order to understand this environmental effect on performance, it would be beneficial to perform this same analysis on physical tests carried out by international rowers on ergometers.

## Practical Application

The method implemented reveals the predominant anthropometric factors that affect each Olympic rowing category. Coupling anthropometry and race pacing is not only helpful to understand which factors work where, but also to improve how athletes' train and perform.

From a practical point of view, this tool can be used to determine what type of rower should be recruited or selected for a boat. Directly for competition, this tool can help coaches and rowers to adapt their strategy according to their morphology and that of their opponents. In the future, it would be interesting to improve this tool by including variables other than morphology. Additionally, it would be possible to individualize the tool for a specific athlete, to understand their strengths and weaknesses for future competitions based on all of their past races.

## Conclusion

In conclusion, this method reveals the predominant anthropometric factors that affect an Olympic rowing category. Coupling morphology and race pacing is not only helpful to understand which factors work where, but also in improving how to train and perform. In addition to helping determine what type of rower should be recruited in a boat, it could also provide significant performance optimization by adjusting factors such as morphotype or racing strategy.

## Data Availability Statement

The original contributions presented in the study are included in the article/Supplementary Materials, further inquiries can be directed to the corresponding author/s.

## Author Contributions

QD, SDe, AL, SDu, J-FT, and AS conceived, designed, performed, and analyzed the research. SDe conceived, designed, and collected data from website. QD and AL carried out the statistical analyzes. QD, SDe, SDu, and AS wrote the manuscript. All authors read, review, and approved the final manuscript.

## Conflict of Interest

The authors declare that the research was conducted in the absence of any commercial or financial relationships that could be construed as a potential conflict of interest.
